# A Highly Productive CHO Cell Line Secreting Human Blood Clotting Factor IX

**Published:** 2018

**Authors:** S. V. Kovnir, N. A. Orlova, M. I. Shakhparonov, K. G. Skryabin, A. G. Gabibov, I. I. Vorobiev

**Affiliations:** Institute of Bioengineering of the Federal Research Centre “Fundamentals of Biotechnology” of the Russian Academy of Sciences, 60-let Oktjabrja Ave. 7, bldg. 1, Moscow, 117312, Russia; M. M. Shemyakin–Yu. A. Ovchinnikov Institute of Bioorganic Chemistry of the Russian Academy of Sciences, Miklukho-Maklaya Str. 16/10, Moscow, 117997, Russia

**Keywords:** blood clotting factor IX, hemophilia B, heterologous protein expression system

## Abstract

Hemophilia B patients suffer from an inherited blood-clotting defect and require regular administration of blood-clotting factor IX replacement therapy. Recombinant human factor IX produced in cultured CHO cells is nearly identical to natural, plasma-derived factor IX and is widely used in clinical practice. Development of a biosimilar recombinant human factor IX for medical applications requires the generation of a clonal cell line with the highest specific productivity possible and a high level of specific procoagulant activity of the secreted factor IX. We previously developed plasmid vectors, p1.1 and p1.2, based on the untranslated regions of the translation elongation factor 1 alpha gene from Chinese hamster. These vectors allow one to perform the methotrexate- driven amplification of the genome-integrated target genes and co-transfect auxiliary genes linked to various resistance markers. The natural open reading frame region of the factor IX gene was cloned in the p1.1 vector plasmid and transfected to CHO DG44 cells. Three consecutive amplification rounds and subsequent cell cloning yielded a producer cell line with a specific productivity of 10.7 ± 0.4 pg/cell/day. The procoagulant activity of the secreted factor IX was restored nearly completely by co-transfection of the producer cells by p1.2 plasmids bearing genes of the soluble truncated variant of human PACE/furin signal protease and vitamin K oxidoreductase from Chinese hamster. The resulting clonal cell line 3B12-86 was able to secrete factor IX in a protein-free medium up to a 6 IU/ml titer under plain batch culturing conditions. The copy number of the genome- integrated factor IX gene for the 3B12-86 cell line was only 20 copies/genome; the copy numbers of the genome-integrated genes of PACE/furin and vitamin K oxidoreductase were 3 and 2 copies/genome, respectively. Factor IX protein secreted by the 3B12-86 cell line was purified by three consecutive chromatography rounds to a specific activity of up to 230 IU/mg, with the overall yield > 30%. The developed clonal producer cell line and the purification process employed in this work allow for economically sound industrial-scale production of biosimilar factor IX for hemophilia B therapy.

## INTRODUCTION

Factor IX (FIX) is the proenzyme of serine protease from the blood coagulation cascade which hydrolyzes the arginine–isoleucine bond in a factor X molecule in the presence of Ca^2+^ and membrane phospholipids to yield activated factor X (FXa). The noncovalent complex of the factors IXa, VIIIa, and X bound to the phospholipid membrane (tenase) is the key element of the positive feedback loop of the coagulation cascade.

The FIX gene resides on the X chromosome. A congenital absence of this gene or a low level of functionally active factor IX cause hemophilia B, the X-linked recessive genetic disorder that occurs in approximately one out of 30,000 males. In particular, the hemophilia among European royal families (Royal family disease) was believed to have been caused by point mutation in the FIX gene, leading to incorrect splicing of its mRNA and the emergence of an inactive, truncated FIX protein [1].

Therapy for hemophilia B was initially limited to periodic transfusions of blood plasma, then later replaced with prothrombin complex concentrates (a mixture of vitamin K-dependent blood clotting factors IX, II, VII, and X). The risk of thrombosis was the key limitation to this therapy. Blood plasma fractionation using the Cohn’s method gave rise to FIX drugs characterized by a higher purity but containing admixtures of activated FIX (FIXa) and other clotting factors: so, the risk of thrombotic episodes persisted. Additional immunoaffinity purification of FIX has made it possible to completely remove these admixtures; however, it did not eliminate the risk of viral or prion infection that exists when other products of blood plasma processing are used. Natural FIX products prepared by purification using immobilized monoclonal mouse antibodies can also cause an allergic response to mouse immunoglobulin and potentially contribute to the emergence of FIX-neutralizing antibodies; i.e., the inhibitor form of hemophilia B [2].

Cloning FIX cDNA [3] and the first successful attempts at producing recombinant FIX in heterologous systems [4, 5] were performed in 1980–1985. In 1986, a FIX producer with an appreciably high specific productivity was obtained on the basis of a CHO cell line [6]. The recombinant FIX product, nonacog alfa (BeneFIX^TM^), was approved for use in the USA and European Union member states in 1997. Production of this drug involves no animal-derived ingredients or donor plasma components. Nonacog alfa is secreted in CHO cells cultured in a medium free of serum or any other animal-derived products. Isolation and purification of FIX involves four chromatographic rounds, without the use of immunoaffinity chromatography. Potentially present viruses are removed by nanofiltration on a filter with a cut-off threshold of 70 kDa.Ready-to-use nonacog alfa is formulated without human albumin [7].

Circulating mature FIX has a molecular weight of ~57 kDa and an average plasma concentration of ~90 nM. FIX consists of four structural domains: the Gla domain, two EGF-like domains (EGF is the epidermal growth factor), and the C-terminal serine protease domain. The N-terminal signal peptide of FIX is cleaved upon translocation of polypeptide into the endoplasmic reticulum; the propeptide directly upstream from the Gla domain is cleaved upon secretion of a mature protein. The activation peptide residing between the second EGF-like domain and the serine protease domain is cleaved at FIX activation. FIX activation in the coagulation cascade is performed by activated factor XI (the intrinsic pathway) or activated factor VII (the extrinsic pathway).

The Gla domain carrying 12 γ-carboxylated Glu residues is located at the N-terminus of a mature FIX molecule. This domain ensures binding of FIX and FIXa to the surface of endothelial cells [8]. The first EGF-like FIX domain contains a high-affinity binding site for a calcium ion and ensures interplay of FIX with factor VIIIa and the tissue factor (factor III). The second EGF-like FIX domain is involved in the formation of the FIXa–FVIIIa–FX complex. It is linked to the serine protease domain via the activation peptide and the single disulfide bond.

The activation peptide FIX contains many post-translational modification sites that affect the properties of FIX, including N-linked oligosaccharides [9]. As a result, the only significant difference between the recombinant FIXa secreted in CHO cells and natural FIXa consists in the level of β-hydroxylation of the Asp64 residue: 0.24 mol/mol in natural FIX versus 0.4 mol/mol in recombinant FIX [8]. Blocking this post-translational modification by inhibitors of 2-ketoglutarate dioxygenase does not substantially alter the procoagulant activity of FIX. The C-terminal domain of serine protease accounts for ~50% of the total weight of FIX; the active site in it is hidden by the activation peptide and becomes exposed once the peptide is cleaved.

Among all post-translational modifications, only γ-carboxylation in the Gla domain is directly responsible for the procoagulant activity of FIX. Nevertheless, the recombinant FIX produced in CHO cells has only one significant difference from the natural FIX used as a drug: recovery of the FIX procoagulant activity in vivo in patients receiving an infusion of recombinant FIX was on average 1.29-fold lower than that for a natural concentrate of human FIX [10]. The reasons for the lower level of procoagulant activity recovery of recombinant FIX are yet to be elucidated, since comparative clinical trials evaluating the pharmacokinetics of nFIX products have shown that recombinant FIX has a longer half-life: 36 h for recombinant FIX versus 32.7 h for natural FIX [11]. The possible reason for the reduced activity recovery of recombinant FIX in vivo can be the fact that there is no Tyr158 sulfation and/or Ser155 phosphorylation within the activation peptide; however, evidence that supports this hypothesis has been obtained only for animal models of hemophilia B [12]

In many cases, the productivity of the systems of heterologous expression of the human FIX gene depends on the ability of the host cell to properly perform its post-translational modification rather than by the level of FIX biosynthesis. In particular, a significant percentage of FIX secreted in CHO cells is inactive at almost any level of specific productivity, since it contains an unprocessed propeptide that completely inhibits the functioning of the Gla domain. Normal processing of FIX propeptide can be restored upon coexpression of subtilisin/kexin-like convertase PACE/ furin or convertase PC5 homologous to it. An optimal level of propeptide cleavage in secreted FIX can be achieved upon coexpression of the truncated variant of human PACE/furin also secreted in the culture medium. Hence, propeptide cleavage can occur not only in the Golgi apparatus, but also in the extracellular space [7].

FIX activity also depends on the degree of γ-carboxylation of the Glu residues in the Gla domain. In fully active FIX, the first 10 Glu residues out of the 12 need to be converted into Gla residues. In a natural FIX molecule, all 12 Glu residues in the Gla domain are fully γ-carboxylated while the degree of modification of the last two residues is reduced in recombinant FIX products, which does not affect the properties of FIX. The conventional method for purifying recombinant FIX by anion-exchange chromatography involving elution with a CaCl_2_ solution at a low ionic strength efficiently removes FIX molecules with a nonfunctional Gla domain. Therefore, an insufficiently high degree of γ-carboxylation of FIX is more likely to affect the yield of the target product rather than the specific procoagulant activity of purified FIX. The total number of Gla residues in purified recombinant FIX secreted in CHO cells can be as high as 11.5 per protein molecule, while specific procoagulant activity is no lewer than 200 IU/ mg, identical to that of the natural protein.

The reaction of γ-carboxylation of Glu residues in vitamin K-dependent protein molecules is driven by vitamin K-dependent γ-glutamyl carboxylase (GGCX [EC 4.1.1.90]) [13]. The reaction takes place in the lumen of the endoplasmic reticulum and precedes proprotein translocation to the Golgi compartment. Dissolved carbon dioxide is a source of the carboxyl group being attached, while the reduced dihydroquinone form of vitamin K (KH_2_ ) acts as an electron donor and a co-factor of GGCX. The reduced dihydroquinone form of vitamin K (KH_2_ ) is converted to quinone 2,3-epoxide (K > O). For the γ-carboxylation reaction, the KH_2_ concentration in the lumen of endoplasmic reticulum needs to be permanently maintained rather high. Reduction of K > O to KH_2_ in vertebrate cells is catalyzed by the VKOR enzyme complex or VKORC (vitamin K oxidoreductase complex), the integral protein (vitamin K 2,3-epoxide-reductase complex subunit 1 (VKORC1 [EC 1.17.4.4]) being its main component) [14].

he attempts at achieving overexpression of the gene encoding human coagulation factor VII (the vitamin K-dependent protein whose Gla domain is functionally active only if all 12 Glu residues are fully γ-carboxylated) in CHO cells showed that the proportion of functionally active FVII molecules is very low and that GGCX overexpression does not increase the specific activity of FVII. Meanwhile, overexpression of the FVII gene in a HepG2 cell line (human hepatocellular carcinoma cells) and BHK (baby hamster kidney) cells derived from the Syrian hamster allows one to produce a predominantly functionally active protein. The varying activity levels of the VKOR complex are the reason why different cells have different abilities to ensure the γ-carboxylation reaction. Overexpression of the human VKORC1 gene in a CHO cell line allows one to obtain a functionally active product and significantly enhance the rate of factor VII secretion [15].

Similar data were obtained for FIX secreted by BHK cells [16]. In the factor IX expression systems in CHO cells limited by the processing level of the propeptide of the target protein, the degree of γ-carboxylation ensured by the endogenous VKORC1 enzyme was sufficiently high to produce a fully functionally active factor IX [7]. Nevertheless, when expression of the FIX gene is significantly upregulated, the degree of γ-carboxylation of Glu residues in the Gla domain can drop, as evidenced by the strong difference in the level of FIX secretion by HepG2 cells (transformed hepatocytes) and cell lines derived from other tissues [17]. HepG2 cells exhibit high VKORC1 activity, thus being more efficient in FIX secretion.

Transfection with a plasmid encoding the known human VKORC1 gene was considered to be the conventional method to enhance vitamin K oxidoreductase activity in cultured cells. However, the relative catalytic efficiency of this method in other mammalian cells is not so evident. VKORC1 orthologs in different mammalian species are not fully homologous; their in vitro catalytic efficiencies differ by approximately fourfold [18]. The protein composition of the VKOR complex is yet to be identified. Thioredoxin-like proteins from the lumen of the endoplasmic reticulum, including TMX, are considered to be the most likely source of electrons for the functioning of VKORC1 [19]. The membrane topology of VKORC1 was determined only by analogy to the bacterial protein from Synechococcus sp. The spatial electron transport chain to cysteine residues in the VKORC1 active site has not been described yet, as opposed to its homolog, VKORC1L1 [20].

We have put forward the hypothesis that the low activity of the VKOR complex in CHO cells can be due to insufficient expression of the gene coding for VKORC1 from Chinese hamster rather than because of its insufficient catalytic efficiency. Therefore, the required maximum level of human FIX secretion in CHO cells can be ensured by coexpressing the target gene, the soluble variant of human PACE/furin, and VKORC1 from Chinese hamster. The target level of expression of the auxiliary genes can be determined by choosing clonal producer cell lines according to the percentage of coagulationally active FIX molecules with a cleaved propeptide. The objective of this study was to produce and characterize these cell lines that secrete FIX.

## EXPERIMENTAL

**Generation of genetic constructs for gene expression**

Generation of the expression vectors p1.1, p1.2-Zeo, and p1.2-Hyg was described earlier in [21]. The DNA fragments encoding the target open reading frames (ORFs) of the human FIX gene, the human or Chinese hamster VKORC1 gene, or human furin and fused with the Kozak consensus sequence (GCCGCCATGG) [22] were produced by PCR using proper adapter oligonucleotide primers. The PCR products were isolated from 1% agarose gel using Wizard SV Gel and PCR CleanUp System reagent kits (Promega, USA) and ligated with a pAL-TA vector (Evrogen, Russia) using DNA ligase from bacteriophage T4 (Fermentas, Lithuania). PCR was performed using oligonucleotide primers and mixtures for the PCR Encyclo PCR kit, Tersus polymerase mix, and ScreenMix-HS (Evrogen, Russia) on a PTC-100 Thermal Cycler (MJ Research, USA). Molecular cloning was performed on a TOP10 Escherichia coli strain (Invitrogen, USA). Plasmid DNA was isolated using a GeneJET Plasmid Miniprep Kit (Fermentas, Lithuania).

The commercially available clone of human FIX cDNA, pCMV6-XL4/NM_000133.2 (sc126517, Origene, USA), and adapter primers AD-9-AbsF and AD-9- NheR (*Table 1*) were used as a source of FIX ORF. The ORF sequence of human VKORC1 was amplified from the pCMV6-XL4/ NM_024006.4 plasmid (sc112318, Origene, USA) with AD-hVKO-AbsIF and AD-hVKONheIR primers.

**Table 1:** The primers used to clone and sequence the expression plasmids

Primer	Nucleotide sequence 5’ → 3’
FIX	
AD-9-AbsF	ttcctcgaggccgccaccatgcagcgcgtgaacatg
AD-9-NheR	atgctagctttcattaagtgagctttg
9SQf	cggtatgtcaactggattaag
9-AS	ctgctggttcacaggactt
VKORC1	
vkof1	gtcgacatgggcaccacctgag
vkof2	gacatgggca ccacctggag gagccc
vkor1	ctcagggccttttggccttgtgttc
AD-CVKO-AbsIF	ttcctcgaggccgccaccatgggcaccacctgg
AD-CVKO-AbsIF	atgctagctcagggcctttt ggcct
AD-hVKO-AbsIF	ttcctcgaggccgccaccatgggcagcacctggggga
AD-hVKO-NheIR	atgctagctcagtgcctcttagccttg
Furin	
AD-FUR-AbsF	ttcctcgaggccgccaccatggagctgaggccctg
AD-FUR-NheR	aatctagactatcactcaggcaggtgtgagggc
IP-fVQ-F	gctgcagagggagcctcaagtacagtggctggaacagcaggtg
IP-fVQ-R	cacctgctgttccagccactgtacttgaggctccctctgcagc
SQ-FUR639-F	caacggtgtctgtggtgtagg
SQ-FUR1228-F	gcccacctcaatgccaacg
SQ-FUR1563-R	cagggtggagcgggtg
SQ-fVQ-R	gttccagccactgtacttg
Primers targeting the vectors	
T7prom	taatacgactcactataggg
SP6	gatttaggtgacactatag
3CH1-Rev	acaaacagttctgagaccg
SQ-5CH6-F	gccgctgcttcctgtgac
IRESArev	aggtttccgggccctcacattg

Total RNA was isolated from 2·10^6^ CHO DG44 cells using TRI Reagent (MRC) in order to obtain the VKORC1 ORF from Chinese hamster. cDNA was synthesized using a Mint kit (Evrogen, Russia) and 2 µl of the RNA template. cDNA was amplified using an Encyclo PCR Kit; the vkof1, vkof2, and vkor1 primers were used to perform PCR of the ORF region. The PCR product was cloned into the pAL-TA vector to yield plasmid pAL-CHOVKORC1. The nucleotide sequence of the inserted fragment was deposited into the GenBank database (accession number, JQ400047.1) on April 3, 2012. The amino acid sequence of the VKORC1 ORF from CHO DG44 cells was deposited into the GenBank database (accession number, AFG26681.1) on April 3, 2012. The PCR product containing the VKORC1 ORF from Chinese hamster with restriction sites for subcloning insertion into an expression vector was obtained using the AD-CVKO-AbsIF and AD-CVKO-NheIR adapter primers and plasmid pAL-CHOVKORC1 as a template.

Human PACE/furin ORF was obtained by PCR using the AD-FUR-AbsF and AD-FUR-XbaR adapter primers and plasmid SC118550 (Origene, USA) as a template. The PCR product containing the ORF of the soluble deletion variant of human PACE/furin protease, with two amino acids deleted (VQ), was cloned into the pAL-TA vector to yield plasmid pAL-Fur. The ORF was then brought in line with the reference sequence NM_002569 by adding six missing nucleotides by inverse PCR using the IP-fVQ-F and IP-fVQ-R primers. Mutagenesis was carried out according to the procedure described in [23], with the following modifications: the primers were phosphorylated with bacteriophage T4 polynucleotide kinase (SibEnzyme, Russia) in bacteriophage T4 DNA ligase buffer (Fermentas, Lithuania) for 30 min at 37°C. PCR was performed using an Encyclo PCR kit according to the following scheme: one cycle for 4 min at 94°C, 2 min at 50°C, and 2 min at 72°C; 11 cycles for 1 min at 94°C, 1 min at 55°C, and 2 min at 72°C. The mixture was diluted twice with normal-strength *Dpn*I endonuclease buffer; 10 AU of this endonuclease was added, and the mixture was incubated at 37°C for 30 min and subsequently at 72°C in the presence of Pfu DNA polymerase (2.5 AU) for another 30 min. The PCR product was purified and ligated. A specific SQ-fVQ-R oligonucleotide was used to search for the regions of altered DNA by colony PCR.

The resulting plasmids pAL-F9, pAL-hVKORC1- AN, pAL-CHOVKORC1-AN, and pAL-FurVQ were sequenced within the insert; the correct ORF areas were cloned into the expression vectors p1.1, p1.2-Zeo or p1.2-Hyg at the AbsI–NheI sites. DNA for transfection was isolated using an EndoFree Plasmid MaxiKit (Qiagen, USA) or a GeneJet™ Midi kit (Fermentas, Lithuania). Prior to transfection, we sequenced the main functional elements of the vectors and repeatedly sequenced the regions of the target ORFs. The plasmids were linearized with PvuI (p1.1 and p1.2-Zeo) or BspHI endonucleases (p1.2-Hygro), precipitated with ethanol, dissolved in phosphate-buffered saline (PBS), and sterilized by filtration through filters with a 0.22 µm pore size (Millipore, USA).

Culturing of CHO DG44 cells (Invitrogen, USA) and transfection with plasmid p1.1-F9 were carried out according to the procedure described in [21]. Forty-eight hours post-transfection, the cells were transferred into a CD CHO medium (Invitrogen, USA) supplemented with 200 nM methotrexate (MTX) and 8 mM glutamine and cultured by passing the cells every 3–4 days until a cell viability above 85% was restored (for a total of ~20 days). The resulting cell population was cloned by limited dilution (1 cell per well) in the MTX-free EXCELL® CHO Cloning Medium (Sigma-Aldrich) supplemented with 8 mM glutamine. The productive clones were identified by ELISA; the selected clones were transferred sequentially into 24-well plates and then, into 6-well plates with the ProCHO 5 culture mixture (Lonza, Switzerland) supplemented with 8 mM glutamine, and cultured in the suspension mode. The most productive cultures were selected by ELISA among the clonal cultures that retained viability upon suspension cultivation. The p1.1-F9-T2/S clone was used for further amplification.

Amplification was carried out in Erlenmeyer flasks containing 30 ml of a ProCHO medium supplemented with 8 mM glutamine and 1, 2, and 4 µM MTX until the cell viability was restored (15–20 days). The cell culture generated in the presence of 4 µM MTX and exhibiting the highest specific productivity was used for the second round of limiting dilution cloning. Clone 3B12 was selected, readapted for suspension cultivation in the ProCHO 5 medium, and used for sequential co-transfection of the linearized plasmids p1.2-Hyg-Fur and p1.2-Zeo-VKORC. The stably transfected populations were selected using hygromycin B and zeocin antibiotics, respectively. The polyclonal population 3B12-FurVC was used to perform final cloning by limiting dilutions according to the procedure described above. The resulting clones were sequentially subdivided into groups according to the expression level of soluble PACE/furin and the level of procoagulationally active FIX. The selected clones were readapted to a ProCHO 5 medium supplemented with 8 mM glutamine and 1 µM vitamin K3 (menadione sulfate, Sigma-Aldrich) and suspension cultivation in Erlenmeyer flasks.

**Real-time PCR**

RT-PCR was conducted using an iCycler iQ real-time PCR system (Bio-Rad, USA) and qPCRmix-HS SYBR master mix (Evrogen, Russia) supplemented with a SYBR Green I intercalating dye. Each reaction was repeated 3 times in a volume of 25 µl in 3–5 replicas. Genomic DNA was isolated using a Wizard SV Genomic DNA Purification System kit (Promega, USA). Total RNA was isolated using an RNeasy Mini Kit (Qiagen, USA). In order to obtain the required amounts of cDNA, we used 1 µg of total RNA and the Mint kit (Evrogen, Russia).

The primers for RT-PCR were selected using the Beacon Designer v7.51 software; primer specificity was tested using the NCBI BLAST tool (http://www.ncbi.nlm.nih.gov/blast/Blast.cgi). Primers not homologous to the nucleotide sequences of Chinese hamster (specific to the FIX region and IRES-DHFR) were applied. The RT-PCR data were processed using the iCycler Iq4 software, including calculation of the reaction efficiency. The copy number of expression cassette integrated into the genome was calculated using a calibration curve plotted for serial dilutions of plasmid p1.1-F9. The PCR results were compared to those of a control amplicon of the *PPIB* gene, which is present only once in the genome of CHO cells, by searching against the NCBI Nucleotide Collection database using the BLAST algorithm

The mRNA expression level was calculated using the relative ΔΔCq method for primers with a known PCR efficiency. The relative increase in the expression level (times) of the gene normalized with respect to the control gene was determined using the formula taken from [24].

**Table 2:** Specific real-time PCR primers

Primer	Nucleotide sequence 5’ → 3’
RT-F9-F	ttagatgtaacatgtaacattaagaatggcag
RT-F9-R	cattaaatgattgggtgctttgag
RT-ID-F	gccacaagatctgccaccatg
RT-ID-R	gtaggtctccgttcttgccaatc
RT-HYG-F	ttcggctccaacaatgtc
RT-HYG-R	gtctgctgctccatacaag
RT-Zeo-F	agttgaccagtgccgttcc
RT-Zeo-R	ggcgaagtcgtcctccac
RT-FURC-F	agcgggacctgaatgtgaag
RT-FURC-R	ggtggttcttctcgatgcca
RT-PPIB-F	gcaggcaaagacaccaatg
RT-PPIB-R	ctccaccttcctcactacatc
RT-bACT-F	gctcttttccagccttcctt
RT-bACT-R	gagccagagcagtgatctcc
RT-cVKOspN-F	aacgggtttgccgtcagaac
RT-cVKOspN-R	cggtaatcctcgtctcgg
RT-cVKOspC-F	gggcttgatgttgcttaatttc
RT-cVKOspC-R	gcaggtgttaggggtaatatg

*Table 2* lists the primers used to assess the copy number of the expression cassette integrated into the genome and the mRNA level.

**Southern blot hybridization**

DNA was biotinylated using a Biotin DecaLabel DNA Labeling Kit (Fermentas, Lithuania). Either plasmid pAL-ID carrying replication initiation domains of the β-lactamase gene identical to those in the expression plasmids p1.1, EMCV IRES, ORF DHFR [21], or the amplification product of plasmid p1.1-F9 from the AD-9- AbsF and AD-9-NheR primers corresponding to FIX ORF was used as a template to generate probes. The genomic DNA to be used for Southern blotting was cleaved with ApaI endonuclease for 16 h, and the DNA fragments were separated in 0.8% agarose gel. The gel was prepared and transferred to a Amersham Hybond-N+ membrane (GE Healthcare, USA) in a buffer with a high ionic strength of 20 × SSC (3 M NaCl, 0.3 M sodium citrate) for 16 h in accordance with the membrane manufacturer’s protocol. DNA was fixed by heating the membrane to 80°C for 2 h. Prehybridization and hybridization were carried out according to the procedure described in [25] in a solution containing 7% sodium dodecyl sulfate, 0.5 M sodium phosphate, and 1% bovine serum albumin (BSA), pH 7.2, for 16 h at 65°C. The membrane was washed according to the manufacturer’s protocol; detection was performed using a Biotin Chromogenic Detection Kit (Fermentas, Lithuania).

**ELISA measurement of FIX concentration**

The FIX concentration was measured using rabbit anti-FIX polyclonal antibodies (LifeSpan BioSciences, USA) (50 ng/well) as an immobilized antibody according to the procedure described in [26]. HIX-1 mouse monoclonal antibodies (F2645, Sigma Aldrich) were used as a specific antibody. The samples, either undiluted or diluted with PBS supplemented with 1% BSA, were placed into the wells. The percentage of FIX molecules with an uncleaved propeptide was measured by ELISA using affinity purified rabbit antibodies targeting the synthetic peptide corresponding to human FIX propeptide as immobilized antibodies according to the procedure reported in [27]. The key ELISA steps were performed as described above.

The procoagulant activity of FIX was determined by activated partial thromboplastin time (APTT) assay using a Factor IX assay (Renam, Russia). Recombinant FIX within the BeneFIX drug (Wyeth, USA) was used as an activity reference standard. The measurements were conducted using a ThromboScreen 400c optical coagulometer (Pacific Hemostasis, USA).

**Measuring furin activity**

Furin activity was determined using a peptide substrate with a detachable 7-amino-4-methylcoumarin moiety Pyr-Arg-Thr-Lys-Arg-AMC (344935, Merck Millipore, USA) according to the procedure described in [27].

**Measuring VKORC1 activity**

VKORC1 activity was measured using DTT as an electron donor according to the procedure described in [28]. The substrate of the enzyme reaction, vitamin K1 2-3 epoxide (K > O), was synthesized from the quinone form of vitamin K1 (Sigma Aldrich, USA) and purified [29].

**Isolation and purification of FIX**

FIX was isolated and purified as follows: the culture medium was loaded into a column packed with the Capto MMC sorbent and equilibrated with a 20 mM sodium citrate solution, pH 7.0, 100 mM NaCl, 0.02% Tween 80; and washed with a 20 mM sodium citrate solution, pH 7.0, 0.1 M NaCl. FIX was eluted with a 20 mM sodium citrate solution, pH 6.5, 200 mM NaCl, 0.5 M arginine, and 0.02% Tween 80. The eluate was diluted fourfold with water, loaded into a column packed with the Capto Q sorbent and equilibrated with a 50 mM Tris-HCl solution, pH 8.0; 100 mM NaCl; washed with a 50 mM Tris-HCl solution, pH 8.0; 200 mM NaCl; and eluted stepwise with the following solutions: 50 mM Tris-HCl pH 8.0; 10 mM CaCl_2_ , and 100–500 mM NaCl. The eluate fractions containing FIX exhibiting full procoagulant activity were diluted twofold with water and loaded into a column packed with the Capto Heparin sorbent and equilibrated with a 50 mM Tris-HCl solution, pH 7.5; 100 mM NaCl. The column was washed with a 50 mM Tris-HCl solution, pH 7.5; 200 mM NaCl. FIX was eluted with a 50 mM Tris-HCl solution, pH 7.5; 500 mM NaCl. The purified FIX solution was concentrated by ultrafiltration using a VivaFlow200 cassette with a 10 kDa PES membrane (Sartorius Stedim, Germany) and transferred into a storage solution containing 8 mM L-histidine, 0.8% sucrose, 208 mM glycine, pH 7.2, and 0.004% Tween 80. The purified FIX solution was divided into aliquot parts, frozen, and stored at temperatures below –70°C.

## RESULTS AND DISCUSSION

**Generation of CHO cells expressing the gene coding for human factor IX**

**Fig. 1:**
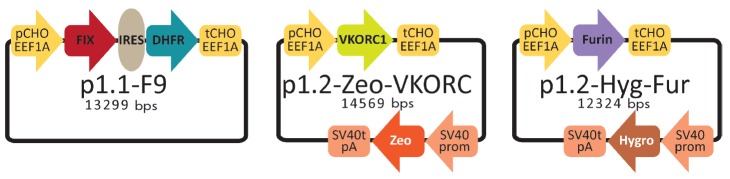
Maps of the expression plasmids p1.1-F9, p1.2-Zeo-VKORC, and p1.2-Hyg-Fur. pCHO EEF1A – functional promoter of the gene of Chinese hamster translation elongation factor 1 alpha, the 5’ untranslated region of this gene and nontranscribing DNA regions flanking this gene; IRES – internal ribosome binding site of the EMCV; DHFR – the open reading frame of the mouse DHFR gene used for selection and genome amplification in eukaryotic cells; tCHO EEF1A – polyadenylation signal, the transcription terminator of the Chinese hamster translation elongation factor 1 alpha gene and the corresponding 3’ nontranscribing DNA region flanking the aforesaid gene; FIX – open reading frame of the human blood clotting factor IX; VKORC – open reading frame of the Chinese hamster VKORC1 gene; SV40 prom – immediate early promoter of the SV40 virus; SV40t pA – transcription terminator and polyadenylation sequence of the SV40 virus; Zeo – open reading frame of the Sh ble gene (*Streptoalloteichus hindustanus *bleomycin) conferring zeocin resistance; Furin – open reading frame of the human PACE/furin protease gene; Hygro – open reading frame of the hygromycin phosphotransferase gene (*E.coli *hpt). Promoter directions are depicted by arrows

The FIX ORF sequence with the inserted synthetic Kozak consensus sequence and a block of stop codons was cloned into the previously designed expression vector p1.1, which was based on noncoding regions of the translation elongation factor 1-alpha of Chinese hamster to yield the expression plasmid p1.1-F9 (Fig. 1).

Long genomic regions flanking the gene encoding translation elongation factor 1-alpha of Chinese hamster ensured the high expression level of the target genes and maintained this level constant during several months of sequential passaging [21]. The ORF sequence of the FIX gene in plasmid p1.1-F9 was linked to the selection marker, murine dihydrofolate reductase (DHFR), by the attenuated internal ribosome binding site of the encephalomyocarditis virus (EMCV IRES), thus ensuring expression of single bicistronic mRNA. This structure provides the strongest possible linkage between the target gene and the selection marker, which is required for amplification of the gene cassettes integrated into the genome of producer cells.

**Fig. 2:**
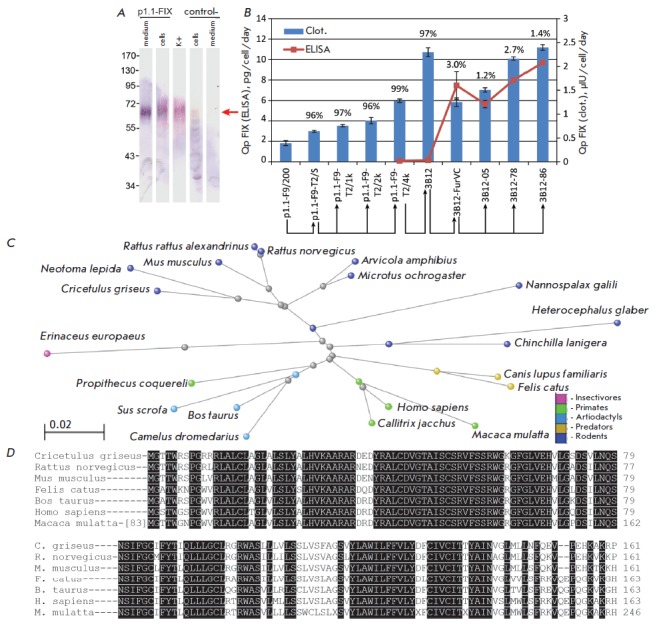
The integrity of the intracellular and extracellular FIX polypeptide chain for the p1.1-F9-T2/S cell line, FIX secretion level and the change in clotting activity for various producer cell lines; the phylogenetic tree for the Chinese hamster VKORC1 protein. Panel *A *– Western blotting of the secreted and intracellular FIX. SDS-PAGE under reducing conditions, detection by polyclonal anti-FIX antibodies, the molecular weight of the marker bands is shown in kDa. Denotation: “K^+^” – recombinant FIX standard; “control-” – untransfected CHO DG44 cells. The mature FIX position is depicted by an arrow. Panel *B *– FIX secretion level and the degree of propeptide processing for cell populations and clonal lines. ELISA-determined specific productivity is shown as bars (left axis). Specific productivity as the clotting activity is shown as a broken line (right axis). The percentage of FIX molecules without the propeptide was determined by ELISA and is shown as numbers above the bars. Specific productivity for both methods is shown as the mean value; error bars represent the standard deviation, n=2. The scheme of production of cell populations and clonal lines is shown with arrows. Panel C – The taxonomic tree for a mammalian VKORC1 protein visualized using the Tree Viewer software (NCBI, USA). The scale bar represents the evolutionary distance measured as the number of substitutions per amino acid residue. Panel D – multiple alignment of the amino acid sequences of VKORC1 variants for the selected mammalian species. Conservative amino acid residues are shown against a black background

Plasmid p1.1-F9 was linearized, with the β-lactamase gene being destroyed, and used to transfect CHO DG44 cells with both *dhfr* alleles being defective. Forty-eight hours post-transfection, the cells were subjected to primary selection in the presence of three different methotrexate concentrations (50, 100, and 200 nM). Stably transfected cell populations were obtained in all three cases; the FIX secretion levels determined by ELISA were 0.69 ± 0.04, 1.05 ± 0.05, and 1.83 ± 0.24 µg/ml, respectively; the population doubling time for the cell cultures was 27–29 h. The maximum FIX titer was detected in the culture obtained in the presence of 200 nm MTX; therefore, immunoblotting of the intracellular FIX and FIX secreted by this cell culture was performed (Fig. 2A). No immunoreactive bands of FIX with an incorrect molecular weight were revealed, thus indirectly indicating that the gene cassettes integrated into the cell genome were not damaged. The resulting polyclonal culture was used to clone cells by limiting dilution. The clonal cell lines secreting FIX with maximum product titer were readapted to the suspension cultivation conditions in a medium with neither nucleotides nor DHFR inhibitors added. The levels of FIX secretion by the three most productive clonal lines after 3 days of cultivation were 11.9 ± 0.4, 12.3 ± 0.4, and 9.9 ± 0.3 µg/ml. The clonal line p1.1-F9-T2/S with specific productivity (Qp) of 2.99 ± 0.06 pg/cell/day and a population doubling time of 22.5 h was chosen for further experiments.

The productivity of the clonal cell line was enhanced by amplifying the target genes in the presence of methotrexate at increasing concentrations. Raising the MTX concentration to 4 µM yielded an oligoclonal cell line with Qp = 5.97 ± 0.18 pg/cell/day, which was used for the second round of limiting dilution cloning. Among the 12 most productive cell clones readapted to suspension cultivation, we selected the clone p1.1-F9-T2/4k-3B12 (referred to as 3B12 in the text and figures below) with Qp = 10.7 ± 0.4 pg/cell/day and a population doubling time of 20.2 h (Fig. 2B).

**Production of the cell lines secreting biologically active FIX**

FIX secreted by the 3B12 clonal cell line was almost completely inactive. The procoagulant activity of FIX in the culture medium was 0.22 IU/ml at a total concentration of 59 µg/ml (determined by ELISA), which corresponded to specific activity comprising 1.8% of that of natural human factor IX. Two reasons are known for the absence of biological activity in FIX: insufficient γ-carboxylation of its Gla domain [7] and retention of propeptide in the secreted FIX molecule so that efficient processing of FIX propeptide by hostcell endogenous proteases belonging to the PACE/furin family becomes impossible [7].

Proper processing of human factor IX propeptide can be ensured by coexpressing the gene encoding signal protease of human PACE/furin. To coexpress the soluble truncated variant of human PACE/furin, we used vector p1.2-HYG that is similar to vector p1.1 but carries the gene coding for hygromycin phosphate transferase under the control of the SV40 promoter (that ensures resistance to hygromycin B) that lies outside the EEF1A gene (Fig. 1).

In order to achieve overexpression of endogenous VKORC1, we cloned the open reading frame sequence of the *vkorc1* gene from cDNA of CHO DG44 cells using primers homologous to the start and end of the ORF of the murine *vkorc1* gene. Sequencing of the cloned PCR product revealed approximately the same level of homology between the *vkorc1* gene from Chinese hamster and the murine and human *vkorc1* genes (Fig. 2C). Unlike its orthologs in other mammals, VKORC1 from Chinese hamster carries a RRR motif [30] flanking the first transmembrane domain and shows much lower homology with the human *vkorc1* gene (Fig. 2D).

The characterized ORF sequence of the *vkorc1* gene from Chinese hamster was deposited into the GenBank database (accession number, AFG26681.1) and cloned into vector p1.2-Zeo to yield plasmid p1.2-Zeo-VKORC1 (Fig. 1). The same expression vector was also used to generate the control construct p1.2-Zeo-hVKORC1 carrying the ORF sequence of the human *vkorc1* gene.

**Table 3:** The VKORC1 activity level in stably transfected cells

Plasmid	Specific VKORC1 activity, % of substrate conversion per 1 mg/ml of total protein in the lysate for 1 h	The relative level of increase in VKORC1 activity, times
Intact CHO DG44 cells	0.38	-
p1.2-Zeo-VKORC1	9.21	24.2
p1.2-Zeo-hVKORC1	3.03	8.0

Note: specific activities were calculated for the linear regions of the curve showing substrate conversion versus total protein concentration in lysates.

Stably transfected polyclonal populations expressing both orthologs of the *vkorc1* gene were produced using CHO DG44 cells. The vitamin K oxidoreductase activity in cell lysate was measured, and the copy number of the integrated expression cassettes was determined. Overexpression of both *vkorc1* orthologs significantly increased the oxidoreductase activity in cell lysate (*Table 3*), while the specific enzyme activity ensured by VKORC1 from Chinese hamster was threefold higher than that ensured by human VKORC1, with the copy numbers of the integrated cassettes being almost equal (5.8 ± 0.3 and 5.5 ± 0.5 copies/genome for the *vkorc1* gene of Chinese hamster and human *vkorc1*, respectively). The transcription levels did not differ significantly for both orthologs: in VKORC1 mRNA from Chinese hamster, it was 0.12 ± 0.03% of the level of β-actin mRNA; in human VKORC1 mRNA, it was 0.09 ± 0.01; P = 0.16. Simultaneously, it was discovered that the highest rate of substrate conversion VK > O by the microsomal fraction of cells transfected with the human VKORC1 gene is approximately 5% per hour, while being at least 9% per hour for the VKORC1 gene from Chinese hamster (data not shown). Since overexpression of the autologous *vkorc1* gene in CHO cells makes it possible to achieve maximum vitamin K oxidoreductase activity, this variant of *vkorc1* was used for the transfection of cell lines secreting FIX.

The clonal 3B12 cells were sequentially transfected with the linearized plasmids p1.2-Zeo-VKORC and p1.2-Hygro-Fur. In the resulting population of stably transfected cells containing three gene cassettes for the expression of factor IX and two auxiliary enzymes, the specific procoagulant activity of FIX in the culture medium was 27% of the reference value; the percentage of secreted FIX molecules with the uncleaved propeptide determined by ELISA was only 3.1% (*Fig. 2B*). Hence, the activity of soluble human PACE/furin in the generated cell population was sufficiently high to ensure an almost complete propeptide cleavage; however, the degree of γ-carboxylation in most cells was insufficient for a proper formation of the Gla domain of FIX. It can be assumed that expression of auxiliary enzymes was not equally efficient in all stably transfected cells. However, unlike VKORC1, soluble PACE/furin secreted by some cells into the culture medium ensured propeptide cleavage in all the secreted FIX molecules.

In order to isolate cells where both auxiliary enzymes exhibit maximum efficiency (i.e., biologically active FIX is produced), we cloned the generated populations by limiting dilutions using the procoagulant activity of the secreted FIX and the peptidase activity level of PACE/furin in the culture medium as a criterion for selecting promising clones. Out of 199 primary cell clones, we chose 80 having maximum FIX concentration. Of those, 24 clones exhibiting the highest PACE/furin activity were selected, eventually yielding 12 clones with maximum procoagulant activity of FIX (data not shown). Five out of the 12 clonal lines were successfully adapted to suspension cultivation in the presence of water-soluble vitamin K3; specific procoagulant activity of FIX in all the clones was > 185 IU/mg. Among these five clones, we identified the main 3B12-86 line with Qp = 11.2 ± 0.3 pg/cell/day and two backup lines, 3B12-78 and 3B12-05.

**Fig. 3:**
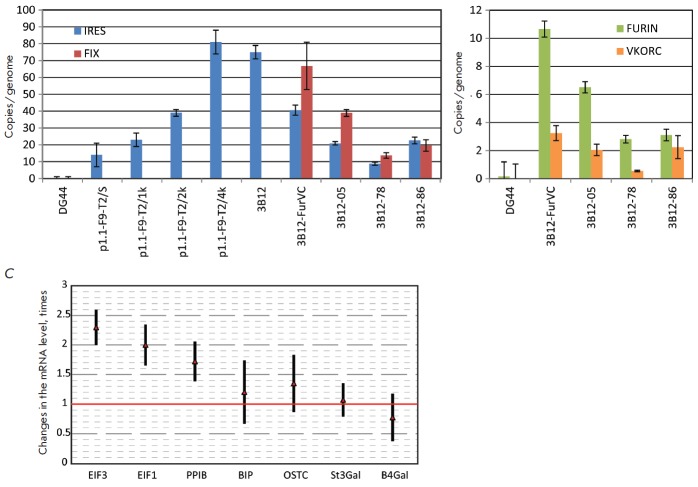
Copy numbers of the target gene and auxiliary genes of furin and VKORC1 in the genome of producer cells; the expression levels of the housekeeping genes for the 3B12-86 cell line determined by quantitative PCR. Panel A – gene copy numbers for the FIX ORF region and the selection marker region (IRES). Denotation: IRES – the amplicon consisting of the IRES fragment and the adjacent DHFR ORF fragment; FIX – the amplicon from the ORF of the FIX gene. Panel B – gene copy numbers for the auxiliary genes of furin and VKORC1. Denotation: FURIN – the amplicon from the ORF of the furin gene; VKORC – the amplicon from the ORF of the VKORC1 gene. Panel C – alterations of the expression levels of the CHO genes involved in translation and post-translational protein modifications in the FIX-secreting cell line as compared to the untransfected control CHO DG44 cells. The results were normalized with respect to the beta-actin mRNA levels. Denotation: EIF1 – eukaryotic translation initiation factor 1a; EIF3 – eukaryotic translation initiation factor 3; PPIB – peptidylprolyl isomerase B; BiP – immune globulin binding protein (Grp78); OSTC – oligosaccharyl transferase complex; St3gal – alpha-2,3-sialyltransferase 3; B4gal – beta-1,4-galactosyltransferase 1. For all panels, the error bars represent a standard deviation (*n*=3–4), one representative experiment out of three runs is shown

The copy numbers of the FIX gene, the sequences of its selection marker and auxiliary genes in the genome of producer lines and parental cell populations were determined by quantitative PCR (*Fig. 3 A, B*). The copy numbers of the FIX and DHFR ORF sequences did not differ significantly in the studied objects; i.e., no signs of fission of the target gene and the gene encoding the selection marker were revealed. In other words, the copy number of the gene encoding the selection marker separately from that of the target gene does not occur despite the amplification of the gene cassettes in the genome of producer cells. When generating the 3B12- 86 cell line, changes in the copy number of the human FIX gene were revealed: the copy number of the FIX gene increased approximately fivefold after amplification, remained constant upon cloning of the amplified population, and subsequently dropped fourfold after plasmids had been cotransfected with the auxiliary genes and repeated cloning had been performed. Meanwhile, the specific productivity of the corresponding cell lines did not decrease. It is possible that once the selection pressure had been eliminated, the low-activity copies of the genetic cassette that had appeared during genomic amplification were rejected.

The copy numbers of the auxiliary genes in all the generated clonal cell lines were significantly lower than that of the FIX gene, because cell clones were not selected in accordance with the maximum activity of the vkorc1 gene. Only one selection round was performed for PACE/furin, and one-third of all produced clones were selected.

In the main producer cell line, 3B12-86, we assessed the changes in the expression levels of several of the housekeeping genes involved in the biosynthesis and post-translational processing of proteins by RT-PCR (*Fig. 3 C*). None of the tested genes showed any significant changes in its expression level, thus indicating that there are no substantial alterations in biosynthesis and protein processing in host cells secreting FIX with the achieved productivity.

**Evaluation of the integrity of the open reading frame sequence in the target gene**

**Fig. 4:**
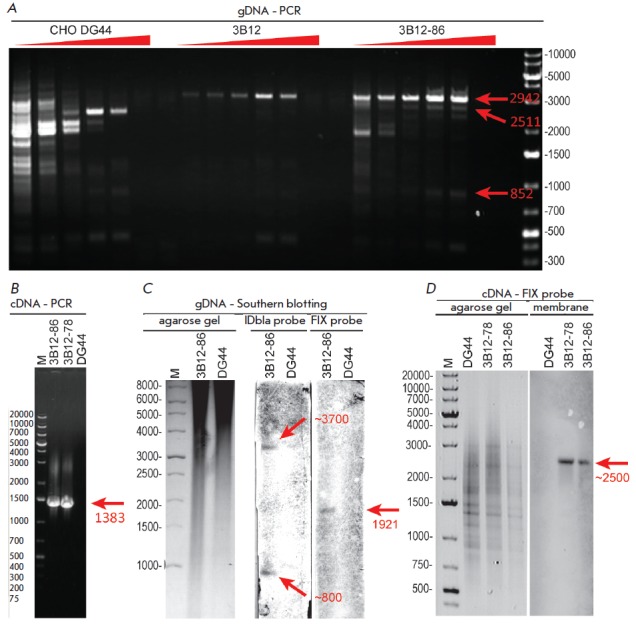
Analysis of target gene integrity in the genomic DNA and cDNA from producer cell lines by PCR, RT-PCR, Southern blotting and pseudo-Northern blotting. DNA ladder bands are shown as bps. Panel A – amplification of whole ORF regions of the target genes in genomic DNA (gDNA) by PCR. The annealing temperature gradient is shown with red triangles; the linear shift in annealing temperature was from 53 to 68 oC. The expected amplification products of the *FIX*, furin, and *VKORC1 *genes are shown with red arrows. Panel B – RT-PCR products for total mRNA with primers towards the 5’- and 3’- ends of the FIX ORF; the amplification product of expected size is shown with a red arrow. Panel C – Southern blotting of gDNA with biotin-labeled probes. The IDbla probe is the probe targeting the IRES and DHFR regions, the ampicillin resistance gene (the *bla *gene) and the region of plasmid origin of replication (ori). The FIX probe is the probe targeting the FIX ORF region. Sizes of the detected target restriction fragments are shown with arrows. Negative agarose gel images; the membrane images were contrast-enhanced to add visibility. Panel D – Analysis of cDNA by pseudo-Northern blotting. Denotation is the same as that in panel C. The actual position and size of FIX cDNA are shown with an arrow

We demonstrated by PCR using genomic DNA and primers specific to the promoter and terminator regions of the employed expression vectors that the predominant PCR products have the correct size (*Fig. 4A*). Namely, a product consisting of 2942 bps corresponding to p1.1-F9 was revealed; amplicons 2511 and 852 bps long corresponding to the ORF sequences of VKORC1 and PACE/furin were also detected for genomic DNA from 3B12-86 cells. Similar amplification of cDNA from the 3B12-86 cell line using primers homologous to the beginning and end regions of the FIX ORF also revealed only a product of the target size (*Fig. 4B*), thus indicating that there are neither long deletions/insertions in the FIX ORF sequence nor mutations that alter splicing of FIX mRNA.

Southern blot analysis of genomic DNA from the 3B12-86 cell line using a probe targeting the FIX ORF sequence revealed one restriction fragment 1921 bps long (*Fig. 4C*), thus indicating that the producer genome does not carry gene cassettes integrated to rupture DNA in the FIX ORF sequence. Southern blot analysis using a probe targeting the regions of plasmid DNA corresponding to the domain of plasmid replication initiation and the sequence of the bla gene found two restriction fragments: a heavy fragment (~3700 bps) approximately corresponding to the calculated cassette integration at the site of its linearization by PvuI restrictase and a short fragment (~800 bps) putatively corresponding to cassette integration involving DNA deletion near the PvuI site. Pseudo-Northern blotting of cDNA produced from the 3B12-86 and 3B12-78 cell lines also revealed only FIX mRNA of the expected length (*Fig. 4D*).

The absence of mutations in the FIX ORF sequence was also confirmed by PCR amplification of the entire FIX ORF sequence from the genomic DNA produced by the 3B12-86 cell line, cloning PCR amplicon, and se quencing of the insert in three plasmid clones. Changes in the FIX ORF sequence were revealed in none of these cases (data not shown).

**FIX isolation and purification**

**Table 4:** FIX purification

Fraction name	FIX:Ag, IU/ml	Fraction volume, ml	FIX:C, IU/ml	FIX:Ag, IU	Total protein quantified by UV spectrophotometric assay, mg	FIX:C, IU	FIX:C/ protein, IU/mg	FIX:Ag/ /protein, IU/mg	Yield of the stage with respect to FIX:Ag	Overall yield with respect to FIX:Ag	Percentage of FIX with propeptide, %
Culture medium	6.04	115	3.6	695	-	414	-	-	-	-	2,6
Capto MMC flow-through and eluate fractions	0.166	273	-	45	-	-	-	-	7%	-	-
Capto MMC eluate	64.79	9	-	583	4.28	-	-	136	84%	84%	-
Capto Q flow-through fraction	0.07	37	-	3	0.69	-	-	4	0.4%	-	-
Capto Q eluate 200 mM NaCl	0.05	12.5	-	1	0.48	-	-		-	-	-
Capto Q eluate 10 mM CaCl_2_	31.75	9.2	25.2	292	1.51	232	154	194	50%	62%	2
Capto Q, 150 mM NaCl + 10 mM CaCl_2_	12.95	10.5	-	136	0.88	-	-	154	23%		
Capto Q 200 mM NaCl + 10 mM CaCl_2_	4.67	10.7	<1	50	0.83	<10.7	<12	60	9%	-	5.8
Capto Q 500 mM NaCl + 10 mM CaCl_2_	13.87	4	-	55	0.57	-	-	98	10%	-	-
Capto Heparin flow-through fraction	0.72	18	-	13	-	-	-	-	3%	-	-
Capto Heparin eluate	36.10	9	38.6	325	1.46	347	237	222	76%	32%	<2

**Fig. 5:**
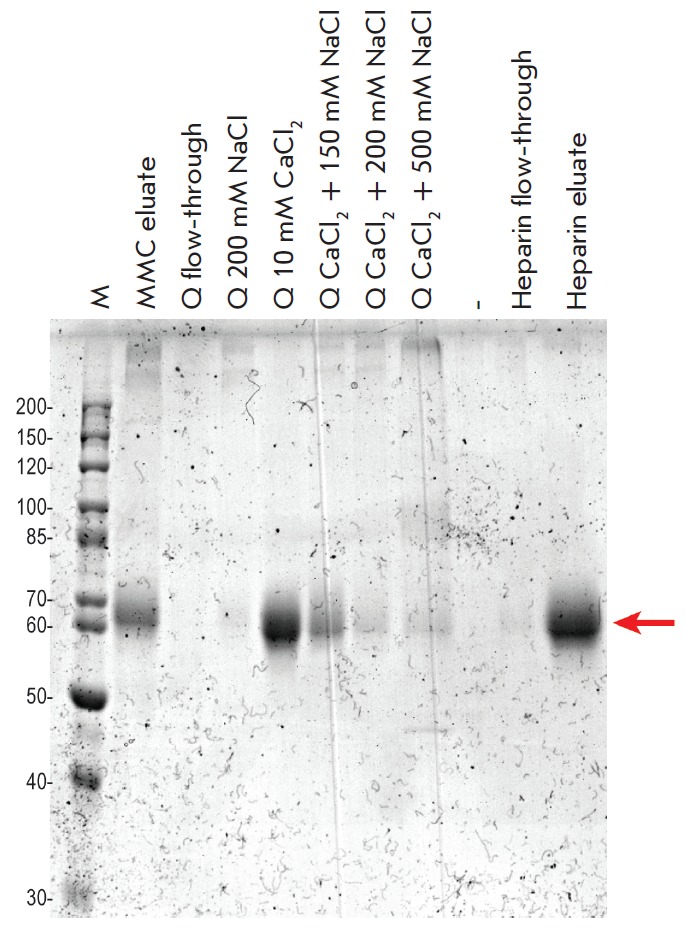
SDS-PAGE analysis of the chromatography fractions obtained during the purification of FIX. Electrophoresis was performed under nonreducing conditions; molecular weights are given in kDa; colloidal Coomassie staining. Denotation: M – marker, eluate – the elution fraction from the corresponding column; flow-through – the flowthrough fractions. The corresponding NaCl concentration in the elution solutions are given for the elution fractions from the Capto Q column; CaCl_2_ concentration was 10 mM for all lanes

FIX was isolated from the conditioned 3B12-86 cell medium and purified using three sequential stages: (1) multimodal chromatography using the Capto MMC sorbent that allows one to isolate FIX from the conditioned medium without any additional preparative stages; (2) pseudo-affinity chromatography utilizing a Capto Q anion-exchange resin and elution of correct IX molecules by a calcium chloride solution with a low ionic strength; and (3) affinity chromatography on a specialized Capto Heparin sorbent, which separates heparin-binding proteins from the remaining molecules. The overall yield of the product was 32%; and the specific procoagulant activity of the purified FIX was > 230 IU/mg, corresponding to that of the known recombinant FIX drug (*Table 4*). SDS-PAGE analysis (*Fig. 5*) showed that most FIX molecules were eluted from the Capto Q sorbent after Ca^2+^ had been added to the low-salt eluent, indicating that these FIX molecules have a properly formed Gla domain whose structural rearrangement upon chelation of Ca^2+^ ions causes elution of Gla proteins from the anion exchanger.

## CONCLUSIONS

The previously developed set of vectors based on untranslated regions of the *EEF1A1 *gene from Chinese hamster can be used to generate cell lines secreting large amounts of functionally active human FIX. The target gene can be amplified in the genome of producer cells by culturing cells in the presence of increasing MTX concentrations, thus enhancing FIX secretion manifold. A sufficient expression level of the auxiliary* vkorc1 *and *PACE/furin *genes can be ensured by cotransfecting the “compatible plasmids” with antibiotic resistance genes. The generated clonal cell lines secreting FIX contain a relatively low copy number of the target gene and only several copies of the auxiliary genes in chromosomal DNA, which may contribute to maintaining the secretion level constant upon longterm cultivation.

A comparative analysis of the overexpressed orthologs of the gene encoding the VKORC1 enzyme has demonstrated that the enzyme activity ensured by the autologous VKORC1 from Chinese hamster is twice as high as that ensured by the artificial *vkorc1 *gene, their copy numbers in the genome being identical. This method of overexpression of the *vkorc1 *gene can be used to generate not only FIX producers, but also other vitamin K-dependent proteins in CHO cells. It seems especially promising to use the *vkorc1 *gene from Chinese hamster to secrete human blood clotting factor VII in CHO cells. The BHK cell line that is currently employed for industrial-scale production of FVII combines a relatively high activity of the VKOR complex and a low total level of FVII secretion. Meanwhile, there are certain limitations to the cultivation mode: unlike CHO, this cell line requires fetal bovine serum to be present in the medium and can grow in a normal manner only under adhesion conditions.

The high specific productivity of the generated cell line secreting blood clotting factor FIX will make it possible to use 4- to 5-day-long plain batch cultivation to produce FIX on an industrial scale; the FIX titer will be ~6 IU/ml. This method of FIX production requires neither using specialized perfusion bioreactors nor elaborating methods to maintain cell culture viability for a long time, making industrial production of recombinant human factor IX much simpler and cheaper.

## Abbreviations

CHO: Chinese hamster ovary cells (Cricetulus griseus)
DHFR: dihydrofolate reductase [EC 1.5.1.3]
EBV: Epstein–Barr virus
EMCV: encephalomyocarditis virus
FIX: clotting factor IX
HAT: a mixture of hypoxanthine, aminopterin, and thymidine
HT: a mixture of hypoxanthine and thymidine
IRES: internal ribosome entry site
MTX: methotrexate
PBS: phosphate buffered saline
VKORC1: vitamin K epoxide reductase complex subunit 1 (EC 1.17.4.4)
APTT: activated partial thromboplastin time
BSA: bovine serum albumin
IU: international unit of factor IX activity (corresponds to the amount of factor IX in 1 ml of pooled plasma from healthy donors)
ORF: open reading frame
RT-PCR: real-time polymerase chain reaction
